# A Novel Ionic Polymer Metal ZnO Composite (IPMZC)

**DOI:** 10.3390/s110504674

**Published:** 2011-04-28

**Authors:** Sang-Mun Kim, Rashi Tiwari, Kwang J. Kim

**Affiliations:** 1 Active Materials and Processing Laboratory, Department of Mechanical Engineering, University of Nevada, Reno, NV 89557, USA; E-Mail: sangmunk@unr.edu; 2 Mechanical and Aerospace Engineering Department, Cornell University, Ithaca, NY 14853, USA; E-Mail: rt275@cornell.edu

**Keywords:** ionic polymer, sensor, photo-luminescence, quenching

## Abstract

The presented research introduces a new Ionic Polymer-Metal-ZnO Composite (IPMZC) demonstrating photoluminescence (PL)-quenching on mechanical bending or application of an electric field. The newly fabricated IPMZC integrates the optical properties of ZnO and the electroactive nature of Ionic Polymer Metal Composites (IPMC) to enable a non-contact read-out of IPMC response. The electro-mechano-optical response of the IPMZC was measured by observing the PL spectra under mechanical bending and electrical regimes. The working range was measured to be 375–475 nm. It was noted that the PL-quenching increased proportionally with the increase in curvature and applied field at 384 and 468 nm. The maximum quenching of 53.4% was achieved with the membrane curvature of 78.74/m and 3.01% when electric field (12.5 × 10^3^ V/m) is applied. Coating IPMC with crystalline ZnO was observed to improve IPMC transduction.

## 1. Introduction

Materials that can sense changes in their environment (external stimuli) and respond in accordance with these changes are classified as “smart” materials. The type of external stimuli (thermal, magnetic, electrical, chemical, mechanical or radiation) and particular responses to these stimuli (reflectivity change, viscosity change, charge generation or stain) leads to smart materials ranging from ceramics to polymers. Recently, smart materials like electroactive polymers (EAPs) [1–5], piezoelectric ceramics [6–8], shape memory alloys (SMA) [9,10], and carbon ––nanotubes (CNTs) [11,12] have been considered for sensors and actuators development. These materials can be used individually or in combinations with each other for integrated sensor-actuator platform development.

Ionic Polymer Metal Composites (IPMCs) have received great attention due to their electroactive nature. IPMCs undergo bending on application of an electric field in its thickness direction (electromechanical transduction) and generate current on mechanical deformation (mechanoelectric transduction). Typically, an IPMC consists of a thin ionomeric membrane sandwiched between metal electrodes. On hydration, cations along with water molecules move towards the cathode on application of an electric field. The migrated cations induce stress in the polymer due to cation-cation repulsion, causing it to bend. On the other hand, bending an IPMC results in discrete regions of high water density causing water molecules and cations to move from their high-density region to their lower density region. This motion of ions inside the polymer produces a current. IPMCs display several advantages like low actuation voltages (1–3 V), flexibility, chemical stability, large actuation displacements (>1 cm) and long life (∼250,000 cycles). The presence of ionomeric membrane results in large capacitance of IPMCs that can be used for energy storage applications.

Since Oguro *et al.* [13] observed a Nafion-based IPMC bending actuator; vigorous research on sensor-actuator systems has been carried out using IPMCs in diverse configurations [14–22]. Despite their inherited transduction properties, IPMCs suffer from small force outputs, nonlinear responses and low current generation capabilities. The degree of transduction is dependent on a variety of factors such as the type of polymer used, counter ion in the polymer, the polymer conductivity, the degree of hydration, the electrode quality and the electrode thickness. Wiring an IPMC sensor/actuator has also been observed to be challenging. Besides, existing measurement techniques increase signal losses in mechanoelectric transduction or interfere with actuation. Many applications could be benefited by a measuring technique that could not only enable wireless measurement of IPMC transduction without hampering its electroactive nature. Current state-of-the-art IPMC actuators/sensors do not possess optical properties that can enable noncontact measurement of IPMC output. The commonly used electrodes like platinum and gold being metals have low surface resistance, but need to be wired for any measurement.

In the reported work, a measuring method obtained by depositing ZnO onto IPMCs, creaing what we have termed as an Ionic Polymer Metal-ZnO Composite (IPMZC), potentially imparts electro-mechano-optical characteristics to IPMCs. IPMZC is based on the principle of photoluminescence (PL) quenching. The process of reduction of PL intensity is called quenching. PL quenching has been observed in semiconductor-metal nanostructure systems. Although the mechanism of PL quenching is not very well understood, the reduction in the PL intensity is believed to be dependant on a number of factors like geometry of the metal, particle size and roughness of the metal surface. The resulting quenching could be due to factors like localized excited state dissociation by electric field, exciton-exciton annihilation and interaction of excited states with charge carriers.

ZnO is one of the widely used functional materials due to its unique optical and piezoelectric properties. ZnO has shown to exhibit a wide direct band gap (∼3.37 eV), a good electrical conductivity (∼5 × 10^2^ *Ω*^−1^*cm*^−1^), and piezoelectric properties [23–25]. The piezoelectric properties of ZnO might be able to convert mechanical energy into electrical energy and such devices could provide potential applications as optical actuators and sensors. ZnO is a good candidate for this application due to its high electrical conductivity and its high PL response in the ultraviolet and visible-wavelength ranges [26–33]. An experimental investigation of the PL (photoluminescence)-quenching [34–40], which is reduction of PL intensity with an increase in applied field or deformation, was performed in the newly developed IPMZC. The attractiveness of this work is the simplicity of the fabrication process and the optical property’s usefulness.

## 2. Experimental Section

### 2.1. Fabrication

The polymer electrolyte membrane used for IPMC fabrication was Nafion 117 (Dupont, 0.18 mm thick, 1,100 g/mol of equivalent weight). Platinization on both sides of the Nafion membrane was carried out by the standard electroless deposition techniques [41,42].

ZnO thin films have been deposited by a number of techniques including DC or RF sputtering [43,44], metal organic chemical vapor deposition (MOCVD) [45,46], molecular beam epitaxy (MBE) [47], pulsed-laser deposition [48,49]. Theses techniques all produce high quality ZnO thin films, which are deposited at relatively high substrate temperatures varying from 200 °C to 800 °C. However, these techniques have serious demerits in case of deposition on IPMCs. The process temperatures are too high to deposit the ZnO thin films without damaging the IPMC and it is quite challenging to obtain ZnO films on both sides of the IPMC. The chemical deposition method to produce thin films from aqueous solutions, at low temperature, presents several advantages with respect to other deposition techniques because of large-scale production capability, simplicity and economy [50–52].

**Scheme 1.**
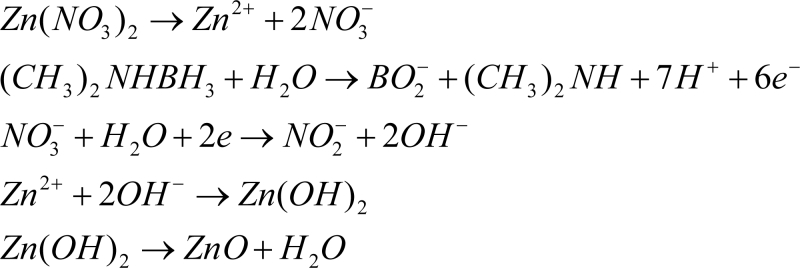
Chemical deposition mechanism of ZnO.

Zinc nitrate, Zn(NO_3_)_2_ and reagent grade dimethylaminoborane (DMAB) were purchased from Sigma-Aldrich. ZnO thin films were synthesized on the Pt IPMC in an aqueous solution composed of 0.1 mol/L hydrous zinc nitrate and 0.1 mol/L DMAB maintained at 60 °C. It has been reported that electrical and optical properties of ZnO film depends on the DMAB concentration [53]. ZnO film prepared from the 0.1 mol/L DMAB solution showed best results [54]. An optimum deposition condition for ZnO film (based on temperature and chemical ratio of Zn(NO_3_)_2_ and DMAB) as reported in [54] was used. The chemical deposition mechanisms are shown in Scheme 1. The nitrate ion undergoes reduction while DMAB is oxidized.

The chemical deposition method for fabrication of IPMZC produces a uniform coating of ZnO on the IPMC substrate. The chances of thermal stress on the IPMC were minimal as the deposition was performed at temperatures below 100 °C.

### 2.2. Characterization

In order to analyze the orientation of ZnO crystals deposited on the electrodes, X-ray diffraction (XRD) patterns of the films were obtained with PANaltical’s X’Pert Pro X-ray diffractometer system with Cu-Kα radiation in the 2*θ* range (30–70°) with a step size of 0.02°. The shapes and sizes of ZnO grains were observed using a scanning electron microscopy (SEM, Hitachi, S-4700) and the compositions of the IPMZC were characterized in cross-sectional view using energy dispersive x-ray spectroscopy (EDS, Hitachi S-4700 equipped with Oxford EDS), respectively. Differential scanning calorimeter (DSC, Q-10, TA instrument) was performed in nitrogen atmosphere with 10 °C min^−1^ heating rate.

**Figure 1.**
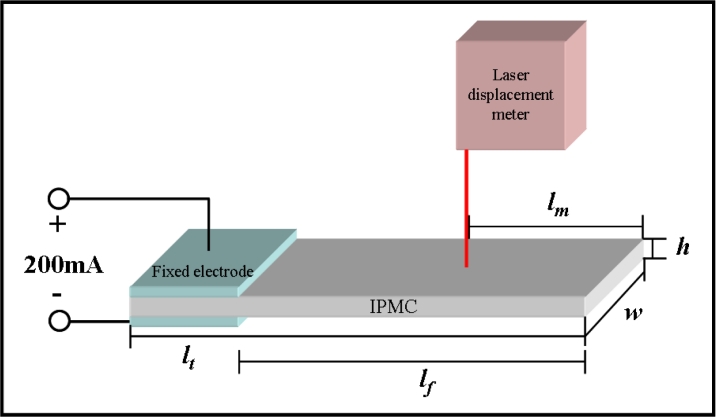
Schematic diagram of IPMC and IPMZC deflection measurement location (*l_t_*: 5 cm, *l_f_*: 4 cm, *w*: 1 cm, *h*: 0.02 cm, and *l_m_*: 2 cm).

The electromechanical and mechanoelectrical properties of the IPMC were investigated by using the potentiostat/galvanostat and shaker systems. IPMZCs were cut into a size of 1 cm width and 5 cm length. The actuation responses of typical IPMCs and IPMZCs were measured using chrono-potentiometry with a square wave for 5 s as a function of the applied current (−200 to 200 mA). Using a laser displacement sensor, the displacement was measured at a distance of 2 cm from the tip of the sample. The experimental set-up is shown in Figure 1.

**Figure 2.**
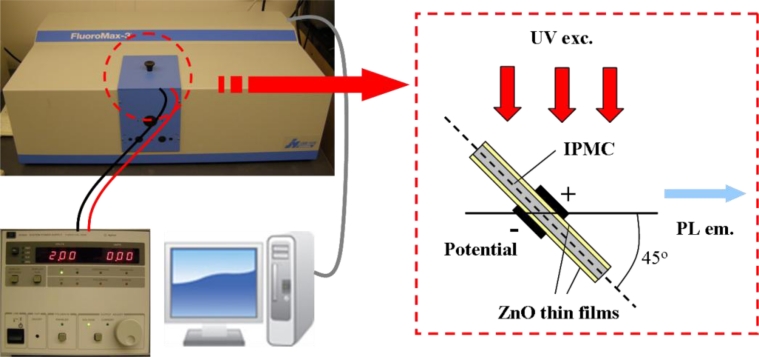
Schematic diagram of the experimental set-up for measuring optical property of IPMZCs. For measurements of PL spectra, the sample is illuminated by UV radiation (280 nm). Spectra are obtained with different potentials from the power supply.

For luminescence investigation, the IPMZC sample was photo-excited at room temperature by an excitation wavelength of 280 nm (xenon arc lamp) in the PL apparatus (FluoroMax-3) and the emission was monitored in the wavelength range 350–500 nm. The opto-electrical property of the IPMZC was characterized with the use of a power supply (potential variation: 0–2.0 V) during the PL measurement as shown in Figure 2. For characterizing opto-mechanical response the IPMZC sample was placed with different curvature while the UV excitation was focused at the center of the sample.

## 3. Results and Discussion

Depositing ZnO onto an IPMC potentially imparts mechano-electro-optical characteristics to the IPMC by integrating the optical properties of ZnO with the tailored electrical properties of an IPMC. The use of an IPMC as the substrate for the ZnO deposition presents certain advantages: (i) the IPMC, as a soft actuator, displays a high deformation with the application of an electric field; (ii) the porous electrodes of the IPMCs enhance the binding of the ZnO on IPMCs; and (iii) the metallic electrodes (Pt or Au) of the IPMC impart a low resistivity and, at the same time, eliminate the need of additional electrodes in a hybrid system. PL quenching in ZnO-Pt nanoparticles has been previously observed [55].

**Figure 3.**
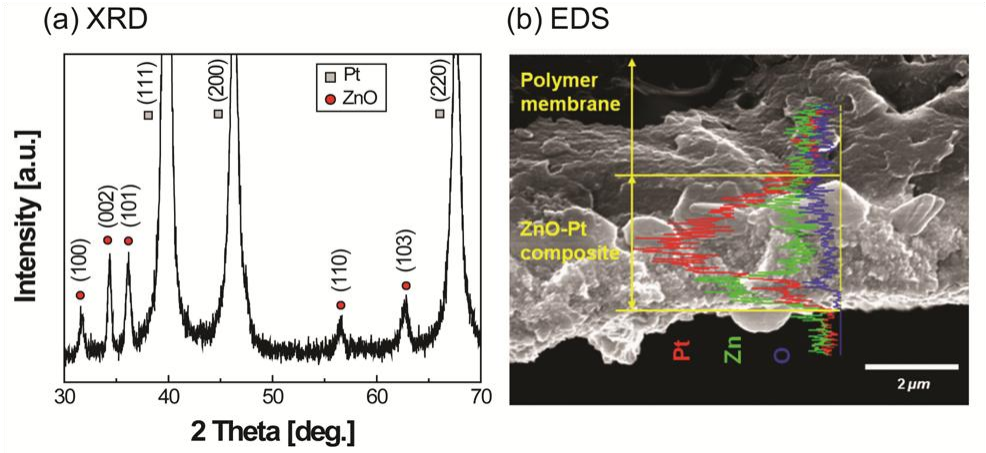

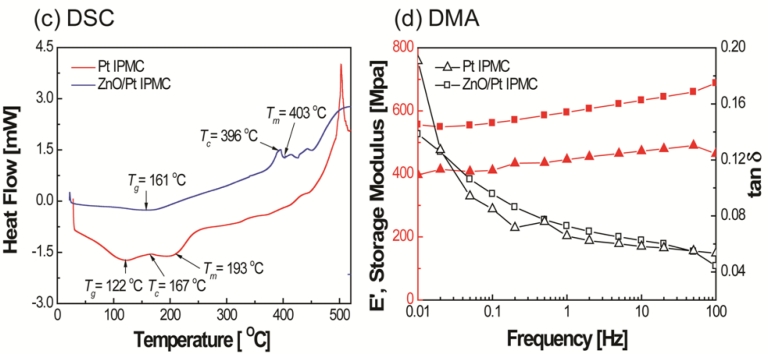
IPMZC characterization results showing ZnO layer on IPMC substrate and change in mechanical characteristics.

The XRD results in Figure 3(a) show all of the diffraction peaks that were assigned to the ZnO. The ZnO had a wurtzite structure and included a preferred growth orientation along the *c*-axis, perpendicular to the substrate.

Although other diffraction peaks were observed, the c-axis (002) plane of the ZnO showed higher intensity than other additional peaks. The XRD diffraction patterns of platinum are from the electrode material of the IPMC. The observed reflections at (111), (200), and (220) are clear and support a crystal structure of metal electrode. From the EDS results in Figure 3(b), the concentration of zinc elements is clearly higher than that of oxygen, indicating that the thin ZnO film is zinc-rich. Most undoped ZnO has *n*-type behavior, which is closely related to an oxygen-deficient non-stoichiometry. The electrical resistivity of the films was strongly affected by the oxygen vacancy and/or zinc interstitials. This mechanism can be easily understood by understanding that the electrical conductivity of the ZnO can be increased proportionally with increasing the oxygen vacancy.

**Figure 4.**
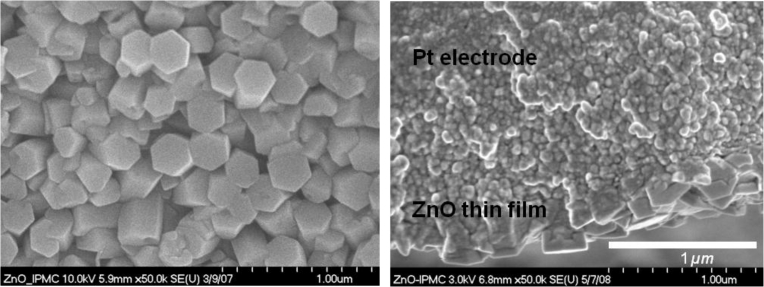
SEM of IPMZC showing hexagonal ZnO crystal on IPMC substrate.

An increase in the melting temperature and storage modulus can be observed from Figure 3(c,d), respectively. This increase is believed to be due to the crystalline layer of ZnO on IPMC. From the SEM image (Figure 4), it was observed that the ZnO crystals grown on an IPMC have a hexagonal shape, and the grains display sizes in the range of 200–300 nm. It can be seen from the cross-sectional view, the thickness of the ZnO thin film ranges between 300–400 nm. However, it should be noted that the ZnO on Pt is susceptible to removal on rubbing or abrasion. Hence care must be taken while working with the IPMZC.

**Figure 5.**
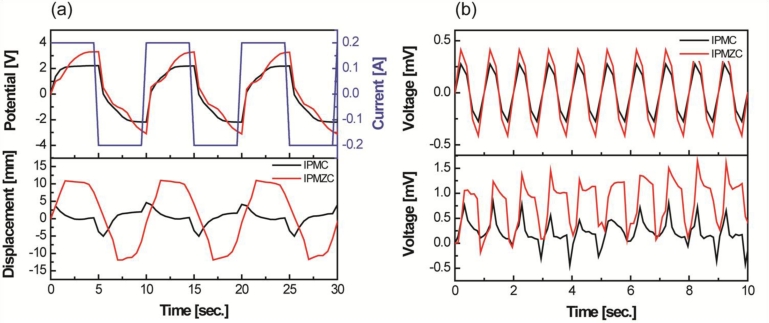
**(a)** Electro-mechanical property: the output potential (top) and corresponding displacement result (bottom) in applied current input (−200 to 200 mA) and **(b)** Mechano-electro property: simulated result (top) and experimental output voltage (bottom) on application of 12.7 mm sinusoidal displacement with the frequency of 1 Hz.

Figure 5 compares the properties of IPMZC with a typical IPMC with a platinum electrode. The output potential results indicate that the IPMZC (−3 to 3 V) responds better electrically with the applied current input (top in Figure 5(a)). According to the corresponding displacement, the IPMZC has a large and stable bending deformation, around 22 mm (peak to peak), which is higher than that of IPMC, shown Figure 5(a).

In addition, the induced displacement of conventional IPMCs shows relaxation behavior, but much less than shown by the IPMZC. This could be due to the combined effect of the piezoelectric property of ZnO and the ionic nature of IPMC, as well as the increase of the conductivity of the “zinc-rich” ZnO thin films. Hence, the displacement on the application of the current becomes more linear. Improvements have been observed in the voltage produced by IPMZC on the application of 12.7 mm sinusoidal displacement, *u*, with the frequency of 1 Hz, as seen in Figure 5(b). These enhancements can be understood by utilizing the piezoelectric [56] and IPMC mechanoelectric equations below [18]. The output of IPMZC is the resultant of the output from the ZnO layer and the IPMC substrate layer. The voltage, in *V*, from ZnO layer is calculated as [57]:

(1) $$V_{ZnO}=\frac{\left(e_{31}\times e_{x}\times t_{ZnO}\right)}{\frac{\left(1+2e_{p}\times t_{IPMC}\right)\times 8.854\times 10^{-12}\times c_{p}}{0.007\times t_{IPMC}}+e_{p}}$$

where *t_ZnO_* and *t_IPMC_* are the thickness of the ZnO and IPMC respectively; *e_p_* is the dielectric constant of ZnO; *e_31_* is the piezoelectric constant for ZnO [58]; and *c_p_* is the capacitive load. The elastic modulus of the IPMZC, *Y_t_* is measured to be 40 × 10^6^ Pa. The average longitudinal strain in ZnO layer with deflection *u* is given as:

(2) $$e_{x}=3Y_{t}(t_{IPMC}+t_{ZnO})\left(l-\frac{1}{100}\right)u$$

The final sensor equation for open circuit voltage is used to calculate the voltage output from the IPMC [18]:

(3) $$V_{IPMC}={\left(\frac{Y_{IPMC}t_{IPMC}^{3}}{2e_{IPMC}L_{\text{free}}^{4}}\right)}^{0.5}u$$

where *e_IPMC_* is the dielectric constant; *Y_IPMC_* is the elastic modulus; and *L_free_* is the free length of the IPMC. The total voltage could be the combination of the above two voltages. From the equations above, one can deduce that the voltage produced by the piezoelectric component is slightly higher than the IPMC (top in Figure 5(b)). Also, due to the crystalline nature of ZnO, there is an observed improvement in the overall property of the IPMC.

The difference in the simulated and experimental data in Figure 5(b) is due to the fact that Equations (1–3) do not take into account the charge transport inside the IPMC and IPMZC. These equations are rather dependent on the sample dimensions, stiffness and overall impedance. Also the effect of interfacial layer between the ZnO and platinum is neglected. Despite these limitations, these equations can be used to predict the trend in the IPMZC output and its dependence on properties like dimensions, stiffness and impedance.

**Figure 6.**
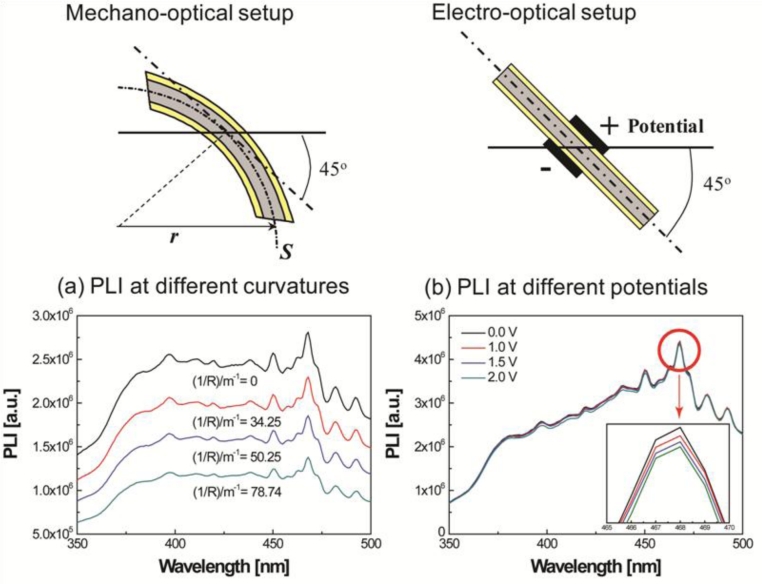

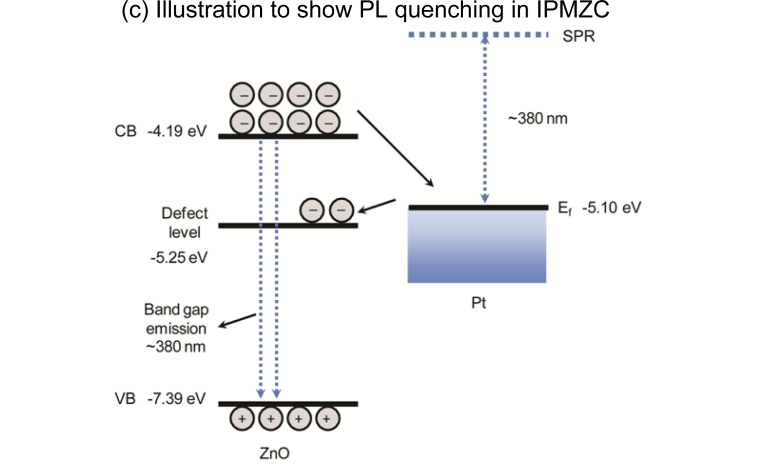
Mechano-optical property set-up and **(a)** PL intensity at different curvatures and, electro-optical property set-up and **(b)** PL intensity at different applied potentials.

The IPMZC sample was photo-excited by an excitation wavelength of 280 nm (xenon arc lamp) in the PL apparatus. The spectra of the sample display a broad emission band with some vibronic structure. A weak emission peak at the wavelength around 384 nm corresponds to the ultra-violet wave near the band edge emission. A relatively strong emission, around 468 nm, has been observed during this process (Figure 6).

The maximum PL intensity was observed when there was no deformation and no electric field present in the IPMZC. In order to explain the PL quenching in IPMZC a schematic diagram (Figure 6(c)) is plotted. As shown in the figure the conduction band of ZnO is located at −4.19 eV and valence band is −7.39 eV. The Fermi level of platinum is at −5.10 eV. A band-gap emission occurs when an electron in the conduction band combines with the hole in valence band. Quenching occurs since the electron in the conduction band of ZnO goes to the Fermi level of platinum which in turn transfers the electron to the defect band of ZnO. This reduces the the band gap in ZnO resulting in decrese in the PL intensity.

**Figure 7.**
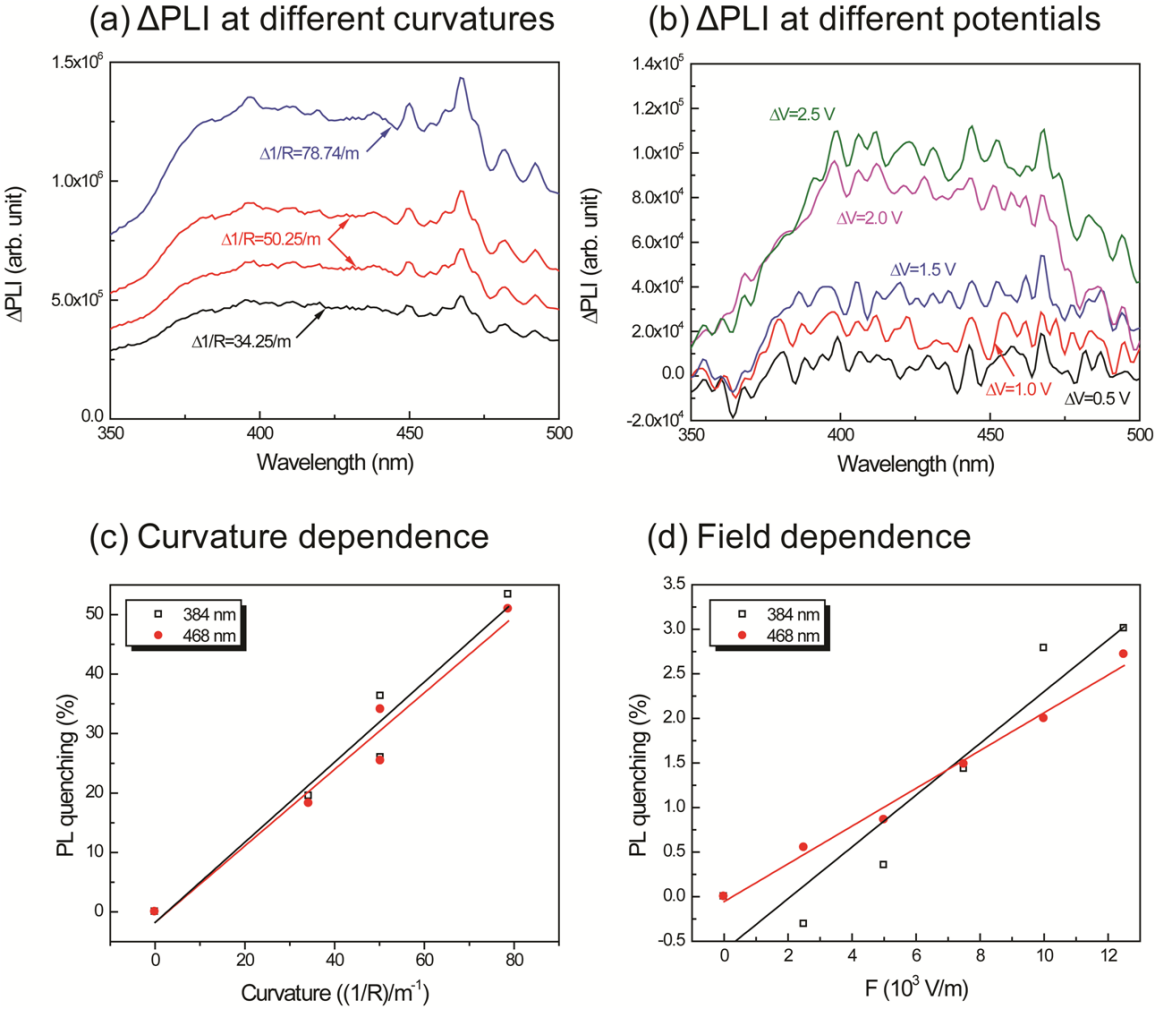
Change in PL intensity with change in curvature **(a)**, and change in input potential **(b)** and respective PL quenching efficiency **(c)** and **(d)**.

In order to clearly observe the effects of curvature and the applied potential, a differential of the intensity was calculated as follows: the intensities measured at different voltages and curvatures were subtracted from the intensity at the initial condition. The operating range of the new IPMZC as 375 nm to 475 nm wavelengths was the result. The quenching efficiency of the mechano-optical and electro-optical properties, $\phi \left(\frac{1}{R}/F\right)$, were calculated, independent of each other, with different curvatures and electric fields applied to the sample and is then defined as:

(4) $$\phi \left(\frac{1}{R}/F\right)=\frac{I_{\lambda }\left(0\right)-I_{\lambda }\left(\frac{1}{R}/F\right)}{I_{\lambda }\left(0\right)}$$

where *I_λ_*(*0*) and *I_λ_*(*1/R / F*) are the PL intensities with and without the imposed curvature or electric field at a particular wavelength, *λ*, respectively. Typical applied potential and curvature values are used for measuring the efficiency. Figure 7 shows these results in detail.

In order to investigate PL-quenching due to the different curvatures of the IPMZC, the sample was bent, and the corresponding intensity was measured (Figure 7(a)). The differential PL intensity and the quenching increased with the curvature. The quenching could be due to the change in orientation or the localized density of ZnO crystals. Analyzing the graph (Figure 7(c)), the quenching efficiency reached a maximum of 53.4% and 50.9% at 384 nm and 468 nm, respectively, while the bending curvature was set to 78.74/m. The electro-optical properties of the IPMZC were also characterized with a potential variation (0–2.5 V) during the PL measurement (inset in Figure 7(b)). Care was taken to as to avoid any bending of the sample on application of voltage across IPMZC. As the electric field increased, the PL intensity decreased. This decrease in intensity with increasing voltage (hence of electric field), can be due to the increase in the depletion region, or dead layer, because of the spatial separation of electrons and holes. The PL-quenching efficiency was measured to be 3.01% at 384 nm when an electric field (12.5 × 10^3^ V/m) is applied across the IPMZC. This efficiency is relatively smaller than that obtained through the bending process, shown in Figure 7(d). Due to its linear dependence on an optical property, associated with the mechanical deformation and the electric field, the IPMZC is an attractive contender as a non-contact type actuation sensor.

## 4. Conclusions

In summary, a novel IPMZC has been successfully fabricated to incorporate the optical and piezoelectric properties of ZnO onto the electroactive nature of an IPMC through chemical deposition technique. The structure of ZnO thin films was observed to be hexagonal in shape, with a grain size of 200–300 nm and average thickness of 300–400 nm. From the EDS result, it was found that the concentration of zinc is higher than that of oxygen, indicating that the ZnO thin film was zinc-rich. The resulting IPMZC exhibits significant transduction property effects. The optical effect of the IPMZC was characterized using photoluminescence. The new IPMC is shown to have working range between 375 to 475 nm wavelengths. The changes in the PL intensity, due to mechanical deformation and an external electric field, can be caused by a variety of reasons: (i) changes in the orientation of the ZnO crystal due to a change in the IPMZC curvature, (ii) changes in the localized density of the ZnO crystal with respect to the imposed strain of the polymer composite, and (iii) variations in the current flow through the high carrier densities in the device.

Further work for the development of an integrated sensor-actuator platform with a ZnO layer for feedback could be performed. In addition, there is need to probe into the effect of grain size and piezoelectric coefficient on the electroactive behavior of IPMZCs.

## Supplementary Materials
