# Supplementary material for: A Novel Ionic Polymer Metal ZnO Composite (IPMZC)
Source: Sensors (Basel). 2011 Apr 28;11(5):4674–87. doi: 10.3390/s110504674 (PMC3231386; doi:10.3390/s110504674)

Supplementary Information

Figure S1. Capacitance measurement.

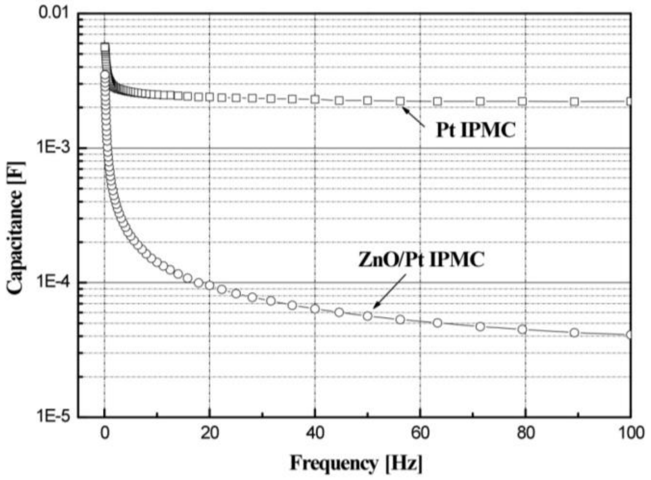

Figure S2. Electro-mechanical property of **(a)** Pt IPMC and **(b)** IPMZC: the out potential (blue) and corresponding displacement result (black) in applied current input (−200 to 200 mA).

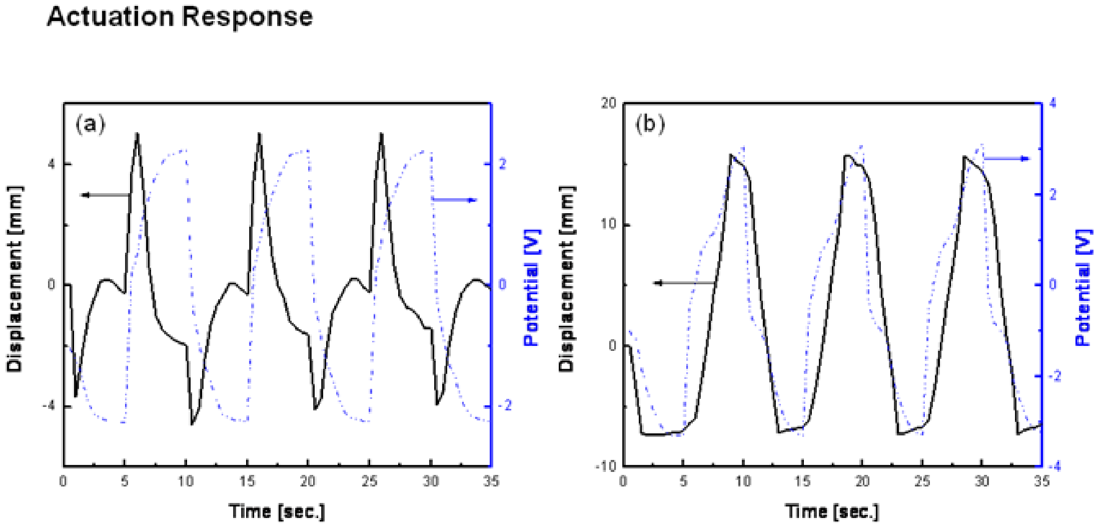

Figure S3. Mechano-optical property of IPMZC: PL intensity (PLI) at the curvature of 50.25/m.

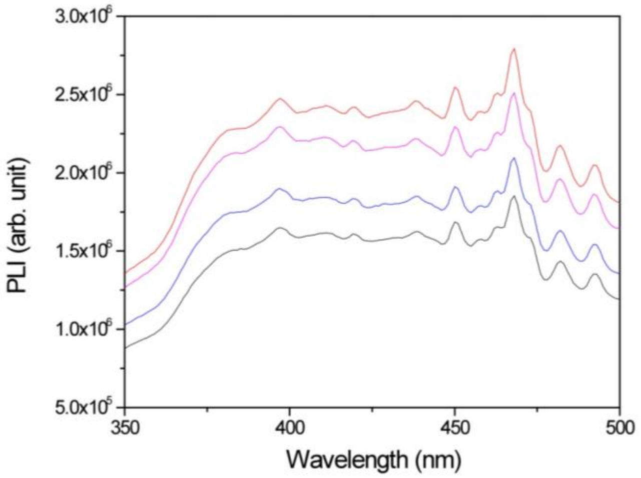

Supplement: Supplementary file 1 [file sensors-11-04674-s001.pdf]
